# Possible Cognitive Dysfunction Associated With High‐Dose Fluoxetine in a Patient on Lithium

**DOI:** 10.1155/crps/5566104

**Published:** 2026-08-21

**Authors:** Adina Tanen, Amy Rajan, Alan Kahn

**Affiliations:** ^1^ Temerty Faculty of Medicine, University of Toronto, 2109 Medical Sciences Building, 1 King’s College Cir, Toronto, ON, M5S 3K3, Canada, utoronto.ca; ^2^ Center for Addiction and Mental Health, 1051 Queen Street West, Toronto, Ontario, M6J 1H3, Canada

## Abstract

Antidepressants, particularly selective serotonin reuptake inhibitors (SSRIs) such as fluoxetine, are widely prescribed medications. Fluoxetine is considered a first‐line pharmacological treatment for common psychiatric conditions such as anxiety, depression, and obsessive–compulsive disorder (OCD). In large clinical trials, fluoxetine has consistently demonstrated a strong safety and tolerability profile over the past 30 years. This case describes a 36‐year‐old Caucasian female with a history of autism spectrum disorder (ASD) and bipolar I disorder treated with lithium, who developed new‐onset cognitive dysfunction following the up‐titration of fluoxetine. Her fluoxetine dose was gradually titrated to 80 mg/day over several weeks, in accordance with dosing guidelines. At that dose, she began experiencing progressive memory loss and confusion, ultimately requiring hospitalization. Extensive evaluation ruled out other potential psychiatric or medical causes of cognitive decline. Upon discontinuation of fluoxetine and reduction of her longstanding lithium dose, her cognitive function improved after 2 weeks and returned to baseline within 4 weeks. While SSRIs are generally well‐tolerated compared to other antidepressant classes, isolated reports suggest fluoxetine may be associated with reversible memory impairment in certain populations. This is the first documented case of reversible memory loss requiring hospitalization associated with fluoxetine in a middle‐aged adult with complex comorbid psychiatric conditions—a demographic frequently prescribed this medication. When prescribing fluoxetine to patients with comorbid psychiatric conditions on lithium, clinicians may consider slow titration and proactive cognitive monitoring.

## 1. Introduction

Antidepressants, particularly selective serotonin reuptake inhibitors (SSRIs), like fluoxetine, are widely prescribed [1], with about one in 10 Americans aged 12 and older taking them. Fluoxetine is considered a first‐line treatment for conditions such as anxiety, depression, and obsessive–compulsive disorder (OCD) and is generally regarded as safe. In large clinical trials, fluoxetine has consistently demonstrated a strong safety and tolerability profile [2, 3]. Over the past 30 years, fluoxetine has continued to be viewed as a safe option.

In addition to improving mood, depressive symptoms, OCD, and anxiety, studies have shown fluoxetine to improve concentration, attention, and cognitive performance in adults [4]. Interestingly, several case reports of adolescents (14–21) and older adults above the age of 60 have documented reversible memory loss associated with its use. Given how often fluoxetine is prescribed to young adults and adults with comorbid psychiatric conditions, it is pertinent to describe rare adverse effects. This is the first case documented of new‐onset cognitive dysfunction requiring hospitalization associated with fluoxetine in a middle‐aged adult on lithium with complex psychiatric comorbidities. Her memory returned to baseline by 4 weeks after fluoxetine discontinuation and a reduction of her longstanding lithium dose.

## 2. Case Presentation

A 36‐year‐old Caucasian woman from a large metropolitan city presented with a complex psychiatric history, including autism spectrum disorder (ASD), OCD, borderline personality disorder, chronic suicidality, bipolar I disorder, and a specific learning disorder. She was living independently in an apartment and previously had held various jobs and achieved postsecondary education. She had multiple psychiatric hospitalizations for comorbid mood disorders and suicidality, with the most recent being 2 years prior.

**Figure 1:**
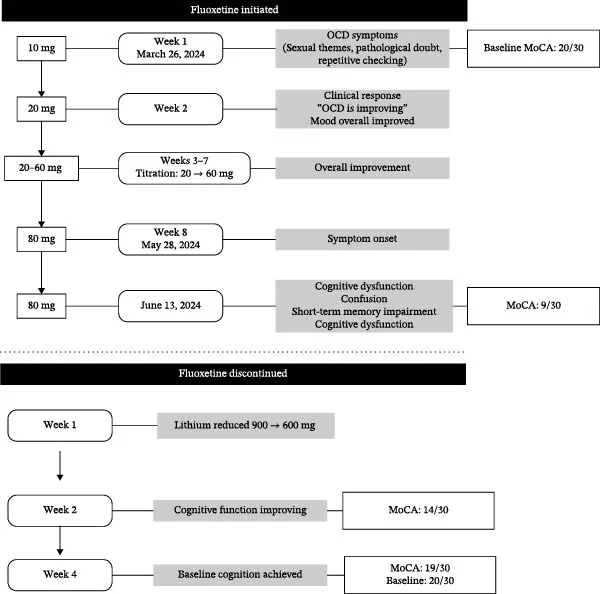
Timeline of fluoxetine dose with associated symptoms and MoCA scores.

The patient was started on fluoxetine by her outpatient psychiatrist 8 weeks prior to the target OCD symptoms, which included sexual themes, pathological doubt, and repetitive checking. The fluoxetine dose was titrated from 10 mg/day starting on March 26, 2024, to 80 mg/day by May 28, 2024 (Figure 1). At a dose of 20 mg, she reported improvement in her OCD symptoms, mood, and anxiety, with a reduction in the intensity of her chronic suicidal ideation. New‐onset cognitive dysfunction and memory loss began shortly after increasing the fluoxetine dose from 60 to 80 mg/day.

On June 13, 2024, she was brought to the emergency department (ED) by her parents due to progressive cognitive dysfunction, confusion, and short‐term memory loss over several weeks. Her family noted increasingly bizarre responses during conversations and disorientation to time, requiring constant redirection to remain focused on tasks. They no longer felt that she could safely live independently due to these concerning symptoms.

In the psychiatric ED, she was unable to provide a reliable history but expressed significant anxiety about her worsening memory. Her thought process was circumstantial and tangential, requiring frequent redirection during the assessment. She was alert and oriented to the person but not the place or time, describing the exam room as located in an “interesting cafeteria.” Initial investigations, including a CT scan and bloodwork, were largely unremarkable. Her serum lithium level was 0.39 mmol/L, her white blood cell count was 10.23 × 10^9^/L, neutrophils were 7.15 × 10^9^/L, thyroid‐stimulating hormone (TSH) 8.12 µU/mL, and vitamin B12 was low at 126 pmol/L. Other labs, including platelets, hemoglobin, electrolytes, liver enzymes, and creatinine, were normal. A longstanding resting tremor was noted, but her neurological exam and gait were otherwise normal. ESR, urea, and copper levels were all within the normal limits.

She scored 9/30 on the Montreal cognitive assessment (MoCA), a marked decline from her baseline score of 20/30 recorded a year prior (Figure 1). She had difficulty with most tasks and frequently needed redirection. The patient expressed anxiety and frustration over her inability to complete the tasks.

The patient was admitted to the hospital for ~4 weeks. A lumbar puncture was performed, yielding slightly elevated WBC but no protein. Her fluoxetine dose was reduced to 60 mg, the last dose before cognitive symptoms worsened.

Careful review of her medications revealed that she was taking aripiprazole 12.5 mg daily, lamotrigine 200 mg daily, lithium carbonate 900 mg daily, and benztropine 1 mg as needed for extrapyramidal side effects. Fluoxetine is the newest addition to her regimen.

The decision was made to discontinue fluoxetine and reduce her lithium dose to 600 mg. Gradually, her cognitive function began to improve. Two weeks later, her MoCA score increased to 14/30. When reassessed 2 weeks later, her MoCA score rose to 19/30, approaching her baseline score of 20/30 from 2023. By this time, her thought processes had become more linear, coherent, and organized. She was able to engage in structured conversations with appropriate responses and no longer required redirection. Her short‐term memory had also significantly improved, and she was fully oriented to time and place. Upon discharge, she nearly regained her normal cognitive function. She was scheduled for follow‐up in a bridging psychiatric clinic and continued long‐term care with her psychiatrist for her comorbid mental illnesses.

## 3. Discussion

**Figure 2:**
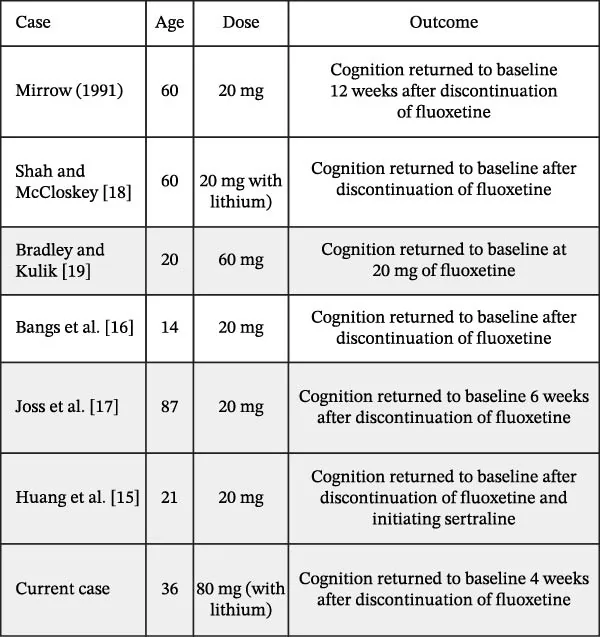
Comparison of past case reports: age, fluoxetine dose, and outcome.

A case of reversible memory loss is described in a middle‐aged female patient with comorbid psychiatric conditions on lithium whose fluoxetine was up‐titrated. The patient’s family began noticing cognitive changes after her fluoxetine dose was increased from 60 to 80 mg. Fluoxetine was the only new medication added to her regimen. Her cognitive function continued to decline until her lithium dose was reduced and fluoxetine was discontinued. Two weeks after stopping fluoxetine, her memory started to improve, and by 4 weeks, it had nearly returned to baseline, as measured by her MoCA scores (Figure 1). Resolution of symptoms within a similar timeframe after discontinuation of fluoxetine is seen in other case reports (Figure 2).

Fluoxetine has long been viewed as a safe antidepressant with tolerable side effects. Compared to tricyclic antidepressants (TCAs), fluoxetine shows lower discontinuation rates due to more acceptable side effects. Common reasons for discontinuing fluoxetine included nausea, nervousness, and insomnia [2]. A similar study conducted by Eli Lilly and Co. in 2000 included 4,016 patients with major depressive disorder (MDD) who were treated with fluoxetine (20 to 80 mg), a TCA, or a placebo. Results again showed that fluoxetine had a lower discontinuation rate compared to that of TCAs, with the most common side effects being asthenia, dizziness, insomnia, nausea, nervousness, somnolence, and tremors [5].

Severe, reversible cognitive impairment requiring hospitalization is rare with fluoxetine. Fluoxetine often does not affect cognition, or it produces cognitive benefits. Ampeuro et al. [6] reviews existing literature, which shows both potential cognitive benefits as well as negative effects of fluoxetine, particularly in younger patients. A study by [7] investigated fluoxetine’s impact on patients with preexisting mild cognitive impairment. After an 8‐week trial involving 44 patients, those on fluoxetine showed *improved* mini‐mental state examination (MMSE) and memory scores compared to those on placebo. In a study by Jaykaran et al. [4], 35 patients on 20 mg/d fluoxetine for 1 month showed statistically significant improvement in attention and concentration, delayed recall, verbal retention, and visual retention on the PGI memory scale. On the other hand, a study by Sayyah et al. [8] showed that SSRI use might be linked to decreased cognitive function during the acute phase of treatment. Fifty treatment‐naive patients with depression or OCD were evaluated using the MMSE while on various SSRIs, including fluoxetine. After 8 weeks, the MMSE scores decreased by an average of 3.28 points. However, the study did not differentiate the cognitive effects of different SSRIs. Bougea et al. [9] found some positive results in their review of fluoxetine’s therapeutic potential as a treatment for slowing the progression of cognitive decline in Alzheimer’s disease, but the results showed low‐to‐moderate evidence. The literature demonstrates mixed evidence, but most often, fluoxetine is associated with positive, or neutral, cognitive effects.

From a pharmacodynamic standpoint, a patient can theoretically experience cognitive dysfunction from the interaction between fluoxetine and lithium. Fluoxetine increases synaptic serotonin through SERT inhibition. Lithium also enhances serotonergic activity by increasing presynaptic serotonin and postsynaptic activities. The symptoms noted in this case, such as tremor and confusion, may have been driven by serotonin toxicity, though there was no evidence of classic serotonin syndrome in this patient. In addition, the symptoms improved rapidly upon discontinuation. From a pharmacokinetic standpoint, lithium is filtered at the glomerulus and reabsorbed in the proximal tubule as is sodium. Fluoxetine has also been shown to cause hyponatremia or SSRI‐associated SIADH [10]. It follows that lithium levels can rise if fluoxetine causes hyponatremia. Family members reported that the patient had poor oral intake, “eating only apples” for weeks prior to symptom onset, which could have theoretically caused mild hyponatremia with volume contraction. However, it should be noted that the lithium level was not elevated, and there was no documented hyponatremia in this case.

Some studies show adverse effects, while others show benefit and augmentation when lithium and fluoxetine are used in combination. Adverse effects of combination use include seizures [11] or induction of mania [12]. Other research actually highlights the benefits of lithium as an adjunct to fluoxetine in treating depression [13–16] and its benefits for cognition in combination use [17]. Studies show that combination use of lithium and fluoxetine causes few, if any, serious adverse effects [18, 19].

Yet, several case reports of young adults (ages 14–21) and older adults (over age 60) have documented reversible memory loss associated with fluoxetine use (Figure 1). Most cases describe memory impairment at a dose of 20 mg (e.g., [20]). For example, one case report detailed cognitive impairment in a 14‐year‐old male on fluoxetine, who exhibited poor memory and forgetfulness on a dose of 20 mg/d [21]. An 87‐year‐old on 20 mg/d of fluoxetine developed progressive memory loss over the course of 6 weeks, which was reversed over 8 weeks once discontinued [22]. A 60‐year‐old college graduate with MDD presented with learning and memory difficulties after initiation of 20 mg/d of fluoxetine [23]. Her memory returned to baseline by 3 months after its discontinuation. Shah and McCloskey [24] are the only other cases in the literature reporting reversible cognitive dysfunction after discontinuation of fluoxetine, where lithium was also prescribed (to a 60‐year‐old patient) and not discontinued. The highest dose reported to be associated with cognitive impairment is 60 mg and involved a 21‐year‐old female with comorbid OCD and disordered eating [25]. No case in the literature documents memory impairment occurring only at a high dose of 80 mg or in a middle‐aged adult. Note that in all cases, resolution of symptoms occurs within weeks upon discontinuation of fluoxetine, as in this case (Figure 2).

While isolated case reports describe reversible cognitive dysfunction with up‐titration of fluoxetine, particularly in younger or older patients and patients in extreme age groups, most large‐scale studies and meta‐analyses show fluoxetine has neutral or beneficial cognitive effects. Therefore, this single case should not be interpreted as a common or dose‐dependent adverse effect but rather suggests caution in highly complex patients with multiple comorbidities and concomitant lithium use.

Confounders of this study include the patient’s comorbid conditions, such as ASD and bipolar disorder. The patient was also taking other medications at the time (aripiprazole, lamotrigine, and benztropine), so polypharmacy is another confounder. Additionally, case reports have an inherently low evidence level due to their scope.

This is a single case with multiple confounders, and causation cannot be firmly established. In this single case, deficits emerged at 80 mg/day, with an improvement in cognition occurring about 4 weeks after discontinuation. Prior case reports have implicated doses as low as 20 mg/day in other populations. However, given the multiple confounders, no firm dose threshold can be established from this case alone. Providers may consider slow titration and proactive cognitive monitoring in patients with complex psychiatric comorbidities on lithium when prescribing high‐dose fluoxetine.

## Funding

The authors received no specific funding for this work.

## Consent

Written informed consent was obtained from the patient for the publication of this case report and any accompanying clinical details. All identifying information was removed to protect patient privacy.

## Conflicts of Interest

The authors declare no conflicts of interest.

## Data Availability

The quantitative data used to support the findings of this study are included within the article.

## References

[1] Brody D. J. and Gu Q. , Antidepressant Use Among Adults: United States, 2015-2018 (NCHS Data Brief No. 377), 2020, National Center for Health Statistics, https://www.cdc.gov/nchs/products/databriefs/db377.htm. 33054926

[2] Pande A. C. and Sayler M. E. , Severity of Depression and Response to Fluoxetine, International Clinical Psychopharmacology. (1993) 8, no. 4, 243–246, 10.1097/00004850-199300840-00006. 8277142

[3] Plewes J. M. , Koke S. C. , and Sayler M. E. , Comparing Time to Onset of Response in Antidepressant Clinical Trials, Statistics in Medicine. (2000) 19, no. 15, 2005–2016. 10.1002/1097-0258(20000830)19:16<2169::aid-sim513>3.0.co;2-o 10931518

[4] Jaykaran , Bhardwaj P. , Kantharia N. D. , Yadav P. , and Panwar A. , Effect of Fluoxetine on Some Cognitive Functions of Patients of Depression, Indian Journal of Psychological Medicine. (2009) 31, no. 1, 24–29, 10.4103/0253-7176.53311. 21938087 PMC3168075

[5] Beasley C. M.Jr., Koke S. C. , Nilsson M. E. , and Gonzales J. S. , Adverse Events and Treatment Discontinuations in Clinical Trials of Fluoxetine in Major Depressive Disorder: An Updated Meta-Analysis, Clinical Therapeutics. (2000) 22, no. 11, 1319–1330, 10.1016/S0149-2918(00)83028-3. 11117656

[6] Ampuero E. , Luarte A. , and Flores F. S. , et al.The Multifaceted Effects of Fluoxetine Treatment on Cognitive Functions, Frontiers in Pharmacology. (2024) 15, 10.3389/fphar.2024.1412420, 1412420. 39081952 PMC11286485

[7] Mowla A. , Mosavinasab M. , and Pani A. , Does Fluoxetine Have Any Effect on the Cognition of Patients With Mild Cognitive Impairment?: A Double-Blind, Placebo-Controlled, Clinical Trial, Journal of Clinical Psychopharmacology. (2007) 27, no. 1, 67–70, 10.1097/JCP.0b013e31802e0002. 17224716

[8] Sayyah M. , Eslami K. , AlaiShehni S. , and Kouti L. , Neuropsychological Effects of Fluoxetine in Patients With Cognitive Impairments, Psychiatry Journal. (2016) 2016, 5480391. 27597949 10.1155/2016/5480391 PMC5002481

[9] Bougea A. , Angelopoulou E. , Vasilopoulos E. , Gourzis P. , and Papageorgiou S. , Emerging Therapeutic Potential of Fluoxetine on Cognitive Decline in Alzheimers Disease: Systematic Review, International Journal of Molecular Sciences. (2024) 25, no. 12, PMID: 38928248; PMCID: PMC11203451 10.3390/ijms25126542. PMC11203451 38928248

[10] Liu B. A. , Mittmann N. , Knowles S. R. , and Shear N. H. , Hyponatremia and the Syndrome of Inappropriate Secretion of Antidiuretic Hormone Associated With the use of Selective Serotonin Reuptake Inhibitors: A Review of Spontaneous Reports, CMAJ: Canadian Medical Association journal = journal de l’Association medicale canadienne. (1996) 155, no. 5, 519–527. PMC1335030 8804257

[11] Sacristán J. A. , Iglesias C. , Arellano F. , and Lequerica J. , Absence Seizures Induced by Lithium: Possible Interaction With Fluoxetine, American Journal of Psychiatry. (1991) 148, no. 1, 146–147, 10.1176/ajp.148.1.146. 1898627

[12] Chouinard G. and Steiner W. , A Case of Mania Induced by High-Dose Fluoxetine Treatment, American Journal of Psychiatry. (1986) 143, no. 5, 686–686, 10.1176/ajp.143.5.686. 3485926

[13] Heninger G. R. , Charney D. S. , and Sternberg D. E. , Lithium Carbonate Augmentation of Antidepressant Treatment: An Effective Treatment for Treatment-Refractory Depression, Archives of General Psychiatry. (1983) 40, no. 12, 1335–1342, 10.1001/archpsyc.1983.01790110077013. 6418110

[14] Lafferman J. , Solomon K. , and Ruskin P. , Lithium Augmentation for Treatment-Resistant Depression in the Elderly, Journal of Geriatric Psychiatry and Neurology. (1988) 1, no. 1, 49–52, 10.1177/089198878800100109. 3150926

[15] Ontiveros A. , Fontaine R. , and Elie R. , Refractory Depression: The Addition of Lithium to Fluoxetine or Desipramine, Acta Psychiatrica Scandinavica. (1991) 83, no. 3, 188–192, 10.1111/j.1600-0447.1991.tb05522.x. 1903237

[16] Pope H. G. , McElroy S. L. , and Nixon R. A. , Possible Synergism Between Fluoxetine and Lithium in Refractory Depression, Journal of Clinical Psychiatry. (1988) 145, no. 10, 1292–1294. 10.1176/ajp.145.10.1292 3262313

[17] Fessel J. , Fluoxetine Plus Lithium for Treatment of Mental Health Impairment in Long Covid, Discover Mental Health. (2023) 3, no. 1, 10.1007/s44192-022-00027-w, 1. 36618714 PMC9810252

[18] Bauer M. , Linden M. , Schaaf B. , and Weber H. J. , Adverse Events and Tolerability of the Combination of Fluoxetine/Lithium Compared With Fluoxetine, Journal of Clinical Psychopharmacology. (1996) 16, no. 2, 130–134, 10.1097/00004714-199604000-00005. 8690828

[19] Fava M. , Alpert J. , and Nierenberg A. , et al.Double-Blind Study of High-Dose Fluoxetine Versus Lithium or Desipramine Augmentation of Fluoxetine in Partial Responders and Nonresponders to Fluoxetine, Journal of Clinical Psychopharmacology. (2002) 22, no. 4, 379–387, 10.1097/00004714-200208000-00008. 12172337

[20] Huang S.-C. , Tsai S.-J. , and Chang J. C. C. , Fluoxetine-Induced Memory Impairment in Four Family Members, The International Journal of Psychiatry in Medicine. (2004) 34, no. 2, 197–200, 10.2190/DY6D-33BP-5WPB-1VDX. 15387402

[21] Bangs M. E. , Petti T. A. , and Janus M.-D. , Fluoxetine-Induced Memory Impairment in an Adolescent, Journal of the American Academy of Child & Adolescent Psychiatry. (1994) 33, no. 9, 1303–1306, 10.1097/00004583-199411000-00012. 7995797

[22] Joss J. D. , Burton R. M. , and Keller C. A. , Memory Loss in a Patient Treated With Fluoxetine, Annals of Pharmacotherapy. (2003) 37, no. 12, 1800–1803, 10.1345/aph.1D154. 14632599

[23] Mirow S. , Cognitive Dysfunction Associated With Fluoxetine, American Journal of Psychiatry. (1991) 148, no. 7, 948–949, 10.1176/ajp.148.7.948. 2053640

[24] Shah A. K. and McCloskey O. , Case Report: Acute Confusional State Secondary to a Combination of Fluoxetine and Lithium, International Journal of Geriatric Psychiatry. (1992) 7, no. 9, 687–688, 10.1002/gps.930070911.

[25] Bradley S. J. and Kulik L. , Fluoxetine and Memory Impairment, Journal of the American Academy of Child & Adolescent Psychiatry. (1993) 32, no. 5, 1078–1079, 10.1097/00004583-199309000-00034. 8407756

